# Regulatory Considerations for the Mother, Fetus and Neonate in Fetal Pharmacology Modeling

**DOI:** 10.3389/fped.2021.698611

**Published:** 2021-07-26

**Authors:** Dionna J. Green, Kyunghun Park, Varsha Bhatt-Mehta, Donna Snyder, Gilbert J. Burckart

**Affiliations:** ^1^Office of Pediatric Therapeutics, Office of the Commissioner, US Food and Drug Administration, Silver Spring, MD, United States; ^2^Office of Clinical Pharmacology, Center for Drug Evaluation and Research, US Food and Drug Administration, Silver Spring, MD, United States

**Keywords:** drug development, fetal, regulatory, pediatrics, Food and Drug Administration, model-informed drug development

## Abstract

The regulatory framework for considering the fetal effects of new drugs is limited. This is partially due to the fact that pediatric regulations (21 CFR subpart D) do not apply to the fetus, and only US Health and Human Service (HHS) regulations apply to the fetus. The HHS regulation 45 CFR Part 46 Subpart B limits research approvable by an institutional review board to research where the risk to the fetus is minimal unless the research holds out the prospect of a direct benefit to the fetus or the pregnant woman (45 CFR 46.204). Research that does not meet these requirements, but presents an opportunity to understand, prevent, or alleviate a serious problem affecting the health of pregnant women, fetuses, or neonates, may be permitted by the Secretary of the HHS after expert panel consultation and opportunity for public review and comment (45 CFR 46.407). If the product is regulated by the US Food and Drug Administration (FDA), FDA may get involved in the review process. The FDA does however have a Reviewer Guidance on Evaluating the Risks of Drug Exposure in Human Pregnancies from 2005 and this guidance does discuss the intensity of drug exposure. Estimation of that exposure using physiologically based pharmacokinetic (PBPK) modeling has been suggested by some investigators. Given that drug exposure during pregnancy will impact the fetus, a number of new guidances in the last 2 years also address inclusion of pregnant women in clinical drug trials. Therefore, the drug-specific information on fetal pharmacology will increase dramatically in the next decade due to interest in drugs administered in pregnancy and with the assistance of model-informed drug development.

## Introduction

In his 1966 treatise on perinatal pharmacology, Sumner Yaffe stated that “The administration of a drug to a pregnant woman presents a unique problem to the physician; not only must he consider maternal pharmacologic mechanisms, but he must also be aware of the fetus as a potential recipient of the drug” (1). This dilemma is still a problem today; how can we assess the effects of a drug administered to the mother on the fetus? For 50 years after Yaffe's publication, researchers and regulators had few options to address this question.

A more recent review of obstetric and fetal pharmacology is available (2), and has led to the development of Obstetric-Fetal Pharmacology Research Centers sponsored by the National Institutes of Health (3). Today we are presented with a unique method of performing a pharmacologic assessment on the fetus without the risk of direct blood sampling. This unique method uses sophisticated modeling, which is being increasingly used in drug development. The critical reasons for this assessment are multiple.

While the teratogenic effects of drugs administered to the mother on the fetus have had a central role in safety assessment since the time of thalidomide, questions are increasingly being ask about the long-term effects of perinatal drug exposure. The potential for this serious consequence of perinatal drug exposure was not lost on Sumner Yaffe 50 years ago. Yaffe and colleagues studied the long-term effects of phenobarbital exposure in the perinatal period on sex hormones in rats, and found that phenobarbital perinatal exposure affected adult rat testosterone levels (4). This research continues today as pharmacoepidemiology studies examine these associations, such as for the maternal use of antidepressants with autism spectrum disorders in children (5). This type of research and its findings are complicated by the exclusion of pregnant women from drug development studies, and the off-label use of drugs in pregnancy (6). Additionally, such post-marketing pharmacoepidemiologic studies require a long time to gain such knowledge. Associations between in-utero drug exposure and long-term outcomes may be able to be addressed by modeling approaches.

Modeling will also assist the development of fetal therapeutics. Knowledge gained from classical approaches of administering medications to the mother intended to benefit the fetus, such as in the treatment of fetal arrhythmias, in conjunction with modeling approaches can be used to advance the science of fetal therapeutics. The antenatal administration of drugs such as corticosteroids to the mother at risk of preterm birth to accelerate fetal lung maturation and prevent neonatal disorders results in highly variable outcomes, and modeling and systems pharmacology may be able to provide consistency to this process. Finally gene and stem cell therapy for the fetus will depend on a high degree of understanding of fetal pharmacology and dosing (7).

These modeling efforts for the fetus can be facilitated by regulatory science and regulatory approaches to requiring and assessing the information generated during drug development. Therefore, the objective of this presentation is to review the ethical and guidance-related regulations and recommendations that affect drug therapy in pregnant women and their fetuses. These current regulations will undoubtedly influence the use of modeling to advance the care of these women and babies.

## HHS Regulations on Research in Mother, Fetus, and Neonate

Regulations to protect individuals in research supported or conducted by the Department of Health and Human Services (HHS) evolved from a series of reports released by the National Commission for the Protection of Human Subjects of Biomedical and Behavioral Research (8) in the 1970s. The Belmont Report (9) or the “Ethical Principles and Guidelines for the Protection of Human Subjects of Research,” the most prominent document issued by National Commission, informed regulations found under 45 CFR 46, subpart A (10), otherwise known as the Basic Policy for Protection of Human Research Subjects as subpart A has been adopted by some federal agencies and is known as the Common Rule. HHS regulations also include three other subparts that are intended to protect specific populations that might be involved in research, including the vulnerable populations of prisoners (subpart C) and children (subpart D) (11). Subpart B, the “Additional Protections for Pregnant Women, Human Fetuses and Neonates (12), is pertinent to the topic of this manuscript.

With the adoption of the New Common Rule in 2018, pregnant women are no longer considered as a vulnerable population under 45 CFR 46.111 (a) (3); nonetheless, the considerations under 45 CFR 46.204 of subpart B (12) still apply. Pregnant women may be included in research approvable by an Institutional Review Board (IRB) only if:

(a) “Where scientifically appropriate, preclinical studies on pregnant animals, and clinical studies, including studies on non pregnant women, have been conducted and provide data for assessing potential risks to pregnant women and fetuses;
(b) The risk to the fetus is caused solely by interventions or procedures that hold out the prospect of direct benefit for the woman or the fetus; or, if there is no such prospect of benefit, the risk to the fetus is not greater than minimal and the purpose of the research is the development of important biomedical knowledge which cannot be obtained by any other means;
(c) Any risk is the least possible for achieving the objectives of the research;
(d) If the research holds out the prospect of direct benefit to the pregnant woman, the prospect of a direct benefit both to the pregnant woman and the fetus, or no prospect of benefit for the woman nor the fetus when risk to the fetus is not greater than minimal and the purpose of the research is the development of important biomedical knowledge that cannot be obtained by any other means, her consent is obtained in accord with the informed consent provisions of subpart A of this part;
(e) If the research holds out the prospect of direct benefit solely to the fetus then the consent of the pregnant woman and the father is obtained in accord with the informed consent provisions of subpart A of this part, except that the father's consent need not be obtained if he is unable to consent because of unavailability, incompetence, or temporary incapacity or the pregnancy resulted from rape or incest;
(f) Each individual providing consent under paragraph (d) or (e) of this section is fully informed regarding the reasonably foreseeable impact of the research on the fetus or neonate;
(g) For children as defined in § 46.402 (a) who are pregnant, assent and permission are obtained in accord with the provisions of subpart D of this part;
(h) No inducements, monetary or otherwise, will be offered to terminate a pregnancy;
Individuals engaged in the research will have no part in any decisions as to the timing, method, or procedures used to terminate a pregnancy; and
(j) Individuals engaged in the research will have no part in determining the viability of a neonate.”

If an IRB cannot approve the research under these provisions, but the IRB determines that the “research presents a reasonable opportunity to further the understanding, prevention, or alleviation of a serious problem affecting the health or welfare of pregnant women, fetuses or neonates,” the IRB may refer the research to the Secretary of the HHS, who after consultation with a panel of experts and a period for public comment, may allow the research to proceed (13).

The FDA is not a Common Rule agency but has parallel regulations for the basic protection of human subjects to those in 45 CFR 46, subpart A. These regulations are found under 21 CFR parts 50 and 56. FDA also has parallel regulations for children found under 21 CFR 50, subpart D. FDA does not have parallel regulations to those under 45 CFR 46, subpart B, for protection of pregnant women, human fetuses and neonates in research. However, any FDA regulated research that is federally funded would be subject to the requirements under 45 CFR 46 as well as the requirements under 21 CFR parts 50 and 56. FDA considers the requirements under 45 CFR 46, subpart B, when reviewing research that includes pregnant women, fetuses and neonates but FDA does not have a formal regulatory process for review of such research.

## FDA Guidances for the Mother, Fetus and Neonate

### Guidance for Industry: Pregnant Women: Scientific and Ethical Considerations for Inclusion in Clinical Trials (April, 2018)

The draft guidance [(14), see Table 1] includes general scientific and ethical considerations to encourage the inclusion of pregnant women in clinical trials when appropriate, noting that the decision to do so necessitates a complex risk benefit analysis that involves both the pregnant woman and the fetus. In addition to studies that might be required to treat pregnancy-specific conditions, the guidance discusses the evaluation of drugs in clinical trials for conditions to treat medical conditions or acute illnesses that are common in women of reproductive potential. These drugs are often used during pregnancy without a clear scientific understanding of the risks and benefits to the mother or to the developing fetus (21). Women should be included in clinical trials because (1) safe and effective treatments are needed during pregnancy, (2) lack of data on dosing, safety and effectiveness of drugs may compromise pregnant women and fetuses, (3) there may be a direct benefit to participation that is not available outside of the research, and (4) limited accessible treatment options for pregnant women is a public health issue. The physiologic changes that occur during pregnancy are unique. Drug pharmacokinetics (PK) and pharmacodynamics (PD) may be altered during pregnancy impacting drug absorption, distribution, metabolism, and excretion (ADME) and consequently, impacting safety and effectiveness (22).

**Table 1:** Summary of FDA guidances for the mother, fetus and neonate.

**Guidance title**	**Month, Year**	**Key contents**	**Reference [this text]**
Guidance for Industry: Pregnant Women: Scientific and Ethical Considerations for Inclusion in Clinical Trials	April, 2018	• General scientific and ethical considerations to encourage the inclusion of pregnant women in clinical trials when appropriate. • Evaluation of drugs in clinical trials for conditions to treat medical conditions or acute illnesses that are common in women of reproductive potential.	(14)
Reviewer Guidance: Evaluating the Risks of Drug Exposure in Human Pregnancies	April, 2005	• Guidance to reviewers for evaluation of human fetal outcome data generated after medical product exposure during pregnancy. • Critical factors to consider when evaluating the effects of drug exposure in human pregnancies, sources of human data on drug exposures, methods for overall assessment of post-marketing human data and labeling.	(15)
Guidance for Industry: General Clinical Pharmacology Considerations for Neonatal Studies for Drugs and Biological Products	July, 2019	• Clinical pharmacology considerations specific to the newborn and emphasizes the need for input from a multidisciplinary team when planning for studies enrolling neonates.	(16)
Guidance for Industry: Post-approval Pregnancy Safety Studies	May, 2019	• Recommendations on how to design investigations to assess the outcomes of pregnancies in women exposed to drugs and biological products.	(17)
Guidance for Industry: Nonclinical Safety Evaluation of the Immunotoxic Potential of Drugs and Biologics	February, 2020	• Immunomodulating potential of drugs and biologicals, and use of ICH guidances	(18)
Guidance for Industry: Safety Testing of Drug Metabolites	March 2020	• Recommended studies for assessing the safety of metabolites such as: general toxicity studies, genotoxicity studies, carcinogenicity studies, and embryo-fetal development toxicity studies.	(19)
Guidance for Industry: Pregnancy, Lactation, and Reproductive Potential: Labeling for Human Prescription Drug and Biological Products — Content and Format	July, 2020	• Recommendations on complying with the Pregnancy and Lactation Labeling Rule (PLLR) to assist with the content and format requirements for 8.1, 8.2, and 8.3 of the USE IN SPECIFIC POPULATIONS subsections.	(20)

As noted earlier, although FDA does not have specific regulations that govern the participation of pregnant women in clinical trials, the general considerations for the participation of individuals as human subjects (23) or as unemancipated minors (24) do apply. Research risks differ based on whether the drug is given as part of clinical care or as a research intervention. In the latter situation, the risk of study participation exceeds minimal risk because of the exposure to the drug whereas an observational study collecting data on a drug administered as part of clinical care might be considered minimal risk (25). A decision to expose the fetus to more than minimal risk includes a determination that the exposure to the drug offers a potential clinical benefit to the mother or to the fetus (12).

FDA considers it ethically justifiable to include pregnant women with a disease or medical condition in a post-marketing clinical trial if there are adequate nonclinical studies to support a clinical trial in pregnant women, there are supportive safety data from nonpregnant women in clinical trials, or from literature or other sources, and if efficacy cannot be extrapolated and/or safety cannot be assessed by other means. In the premarket setting, pregnant women may be included in clinical trials if there are adequate nonclinical data to support study in pregnant women and the study intervention holds out a prospect of direct benefit to the mother or fetus, and the pregnant woman has not responded to other treatment options or the study interventions are not available outside of the research setting. Pregnant women with severe disease with limited treatment options may be the most appropriate for clinical studies. PK data should be collected in these studies; data from phase two studies can be used to guide dosing in phase 3. Drug exposure in the fetus/newborn can be assessed by collection of cord blood or from the neonate at the time of delivery, depending on drug exposure and the half-life of the drug. Safety monitoring in any trial where pregnant women will take part should include adequate obstetrical and perinatal expertise in order to recognize safety concerns unique to the pregnant woman and the fetus.

If a woman becomes pregnant during a clinical trial, un-blinding should occur and the risk and benefits of continued treatment with the investigational product should be reviewed. A woman may continue in a clinical trial and receive investigational treatment if the benefits of treatment outweigh the risks of continued fetal exposure vs. transition to other treatment options. Informed consent should be obtained for continued study participation. These situations offer an opportunity to collect steady state PK data in the pregnant woman to inform drug modeling and simulation (14, 26) and dosing during pregnancy. The outcome of the pregnancy should be recorded regardless of whether the woman continues to participate in the study.

### Reviewer Guidance: Evaluating the Risks of Drug Exposure in Human Pregnancies (April, 2005)

Despite the lack of information on the safety of drug use during pregnancy, most pregnant women likely will be exposed to drugs. Knowledge of teratogenic potential is a critical part of a drug's benefit/risk profile. However, pregnant women are rarely included in clinical trials. Currently, majority of the data on teratogenicity are derived from inadvertent pregnancy exposures during clinical trials of new products, fetal exposure occurring before a woman knows she is pregnant or from some women who enter pregnancy with medical conditions that require continuing drug therapy. Such data are usually insufficient to permit an adequately powered statistical analysis.

The guidance on Evaluating the Risks of Drug Exposure in Human Pregnancies developed in 2005 [(15), see Table 1] is aimed at guiding reviewers to evaluate human fetal outcome data generated after medical product (including drug and biological products including vaccines) exposure during pregnancy. The guidance describes critical factors to consider when evaluating the effects of drug exposure in human pregnancies, sources of human data on drug exposures, methods for overall assessment of post-marketing human data and labeling of such products. This guidance should be used in conjunction with more recent guidances, such as the Reproductive and Developmental Toxicities—Integrating Study Results to Assess Concerns Guidance for Industry (27).

Critical factors to consider during evaluation of a product for teratogenic potential include consideration of background prevalence of adverse pregnancy outcomes, combined vs. individual rates of birth defects, major vs. minor birth defects, timing and intensity of exposure, variability of response and class effects. Typically, a drug must cross the placenta and reach the fetus in sufficient concentration to cause an effect. Most teratogens have a threshold below which adverse effects do not occur. Conversely, almost all exposures can be toxic to the fetus if the dose is high enough, even if only indirectly through maternal toxicity. Dosing, including frequency and duration of exposure, is therefore an important consideration in fetal drug exposure. This guidance does not discuss the detailed methodologies for estimating in-utero intensity of drug exposure possibly due to its publication at a time when physiologically based pharmacokinetic (PBPK) modeling was still in its infancy. Recent developments in PBPK models of pregnancy for understanding maternal-fetal drug transfer look promising (28). However, these models need significant refinement before they can be used routinely in drug development to predict intensity of fetal exposure during maternal fetal drug transfer.

Information on human gestational drug exposures will emerge during the post-marketing phase for virtually all drug products. Evidence from all sources, including human data from case reports, epidemiology studies, and animal data, should be considered collectively to determine the strength of the relationship between drug exposure and teratogenicity. Data from embryo-fetal developmental toxicity studies of drug metabolites in animals must also be considered (19).

The only data on fetal effects initially available in the product labeling usually comes from animal reproductive toxicology studies. As part of the Periodic Safety Update Report (PSUR) sponsors are asked to specifically report on “positive or negative experiences during pregnancy or lactation,” by evaluating new human data as they become available, in the context of what is already known about the reproductive effects of the drug, and, if clinically relevant, communicate conclusions regarding risk or lack of risk associated with gestational exposure in the product labeling.

### Guidance for Industry: General Clinical Pharmacology Considerations for Neonatal Studies for Drugs and Biological Products (July, 2019)

The neonatal population is a highly heterogenous patient group that has historically been understudied in clinical research. FDA-approved product labeling is often devoid of neonatal-specific information on drug dosing, safety and efficacy, and most drugs administered in neonatal intensive care units (NICUs) are used off-label. As such, when treating this vulnerable population, health care professionals frequently must rely on professional judgment to inform their clinical decision-making. In order to gain the needed information on the safety and efficacy of medications used in neonates, it is imperative to encourage their inclusion in clinical research, as well as encourage the development of new therapies for conditions unique to the newborn. In response to a provision included in the FDA Reauthorization Act (FDARA) of 2017, FDA published a draft guidance on general clinical pharmacology considerations for neonatal studies [(16), see Table 1]. The draft guidance discusses clinical pharmacology considerations specific to the newborn and emphasizes the need for input from a multidisciplinary team when planning for studies enrolling neonates.

Similar to the International Council for Harmonization (ICH) E11 addendum (29), this draft guidance defines the neonatal period for the term and post-term newborn as the day of birth plus 27 days, and for the preterm newborn as the day of birth, through the expected date of delivery plus 27 days. It also describes subgroup classifications for the neonatal population [e.g., based on gestational age, postnatal age (PNA), post-menstrual age (PMA), birth weight] and notes the importance of considering stratification as a means for defining more homogenous groups of neonates in a trial. Compared to adults and older children, neonates exhibit unique ADME characteristics. Drug ADME in the neonate can be affected by body size, growth/maturation trajectories, underlying illness and concomitant medications which can result in inter- and intra-individual variability in PK measures. Evaluating products in neonatal studies that include a wide spectrum of PMA and PNA subgroups can help to account for this variability.

Characterization of the PK and PD of a drug can inform rational dosing recommendations for the neonatal population if the ontogeny of factors affecting ADME is considered (30, 31). It is important to leverage all existing PK and PD data from other populations (e.g., adults and other pediatric subgroups) to help determine an initial dose for neonatal studies. Quantitative approaches, such as modeling and simulation, can have utility in helping to predict neonatal doses and optimize clinical trial designs. When designing neonatal studies, sparse sampling is a practical approach for obtaining PK data; opportunistic and scavenged sampling can also be considered. For analysis, a previously developed population PK model in an older population can be redeveloped using the newly acquired neonatal data to create a PopPK model that is applicable for neonates to adults. In the absence of prior neonatal data for which a model is built, sparse data can be used to confirm a neonatal PBPK model that has been appropriately scaled to neonates or a population PK model that has incorporated expected changes in growth and maturation on PK parameters. Age-appropriate formulations are required for neonatal studies and safety data should be obtained.

### Guidance for Industry: Post-approval Pregnancy Safety Studies (May, 2019)

The purpose of this draft guidance [(17), see Table 1] is to provide recommendations on how to design investigations to assess the outcomes of pregnancies in women exposed to drugs and biological products. Section 505(o) (3) of the Federal Food, Drug, and Cosmetic Act (FD&C Act) authorizes FDA to require certain post-marketing studies or clinical trials for prescription drugs. The goal of post-approval pregnancy safety studies is to provide clinically relevant information about the use and safety of the products during pregnancy, through inclusion of the information in a product's labeling. This guidance describes three general approaches that can be used in the post-marketing setting to evaluate the product safety during pregnancy:

- Pharmacovigilance—Case reports have been most useful and influential in situations where the adverse pregnancy outcome rarely occurs. Examples include: isotretinoin (32), and oligohydramnios with trastuzumab (33). However, it remains challenging to determine whether a causal relationship exists between a product exposure and an adverse pregnancy outcome. Therefore, observational studies such as pregnancy registries usually are needed to provide additional information.
- Pregnancy Registries—A pregnancy registry actively collects information on product exposures during pregnancy and associated pregnancy outcomes by enabling voluntary participation of women who have been exposed to a specific drug of interest. While it is useful to collect data on the effects of rare exposures during pregnancy, it alone may not be sufficient to assess the safety of products, due to challenges of achieving sufficient enrollment. Use of complementary studies with different study designs may help address these limitations and provide greater confidence in the conclusions.
- Complementary Studies—Additional studies that complement data obtained from pregnancy registries and other sources can be implemented to better understand the specific effects of a product during pregnancy, and to more precisely quantify the magnitude of an association between a pregnancy exposure and a specific outcome.

### Guidance for Industry: Nonclinical Safety Evaluation of the Immunotoxic Potential of Drugs and Biologics (February, 2020)

This draft guidance [(18), see Table 1] deals with the immunomodulating potential of drugs and biologicals, and makes extensive use of ICH guidances such as:

- S8 Immunotoxicity Studies for Human Pharmaceuticals (April 2006)
- M3(R2) Nonclinical Safety Studies for the Conduct of Human Clinical Trials and Marketing Authorization for Pharmaceuticals (January 2010)
- S6(R1) Preclinical Safety Evaluation of Biotechnology-Derived Pharmaceuticals (May 2012)
- S5(R3) Detection of Toxicity to Reproduction for Human Pharmaceuticals (November 2017)

Since the immune system is a very complex and highly regulated network, the assessment of the potential toxicity of a new drug or biologic agent is difficult to characterize. This guidance stresses the “weight-of-evidence” approach for general immunotoxicity assessments, as discussed in ICH S8.

Immune suppression or stimulation could potentially produce deleterious effects on the mother and fetus. Immunostimulation is a particular concern, in view of the previous experience with cytokine release due to the monoclonal antibody TGN 1412 (34). There are now *in vitro* assays that can assess this risk, and the expectation is that these cytokine release and immune activations assays will be conducted to establish the effective concentration (EC) values such as EC_20_, EC_50_ and EC_80_. Additional studies of antibody-mediated immune stimulation, autoimmune reactions, or effects on innate immunity may be necessary.

In some cases, more extensive testing with developmental animal studies may be warranted. These studies may be necessary in situations where the drug product has been shown to elicit immunotoxicity in nonclinical studies with adult animals; the drug or drug class is known to directly affect the immune; or there is reasonable evidence that the mechanism of action or the pharmacology of the drug product could affect the developing immune system. If an evaluation of existing nonclinical toxicity studies indicates the potential for enhanced toxicity in pediatric patients, juvenile animal studies should be considered for products being developed in some therapeutic indications.

### Guidance for Industry: Safety Testing of Drug Metabolites (March 2020)

Drug metabolites may need to be determined in nonclinical studies when there are disproportionate drug metabolites, that is, metabolites identified only in humans or present at higher plasma concentrations in humans than in any of the animal species used during standard nonclinical toxicology testing. It is not standard practice for drug metabolites to be evaluated separately in a cross-species safety assessment. As a result, their specific contribution to the overall toxicity of the parent drug has often remained unknown. Technological advances, however, have greatly improved the analytical abilities to detect, identify, and characterize metabolites and may allow a better understanding of the role metabolites play in drug safety assessment. This guidance [(19), see Table 1] describes recommended studies for assessing the safety of metabolites such as: general toxicity studies, genotoxicity studies, carcinogenicity studies, and embryo-fetal development toxicity studies. It notes that embryo-fetal development toxicity studies with the drug metabolite are required when a drug is intended for use in a population that includes women of childbearing potential, and that the FDA may ask for other reproductive toxicity studies on a case-by-case basis, depending on these study results.

### Guidance for Industry: Pregnancy, Lactation, and Reproductive Potential: Labeling for Human Prescription Drug and Biological Products — Content and Format (July, 2020)

On December 4, 2014, the FDA published the final rule “Content and Format of Labeling for Human Prescription Drug and Biological Products; Requirements for Pregnancy and Lactation Labeling,” referred to as the pregnancy and lactation labeling rule (PLLR). This draft guidance [(20), see Table 1] provides recommendations on complying with the PLLR to assist with the content and format requirements for the 8.1 Pregnancy, 8.2 Lactation, and 8.3 Females and Males of Reproductive Potential of the USE IN SPECIFIC POPULATIONS subsections.

- 8.1 Pregnancy—This subsection contains information on what is known about the drug's effect on pregnancy, including labor and/or delivery, and the availability of a pregnancy exposure registry. The information about Clinical Considerations for this subsection can include: Disease-Associated Maternal and/or Embryo/Fetal Risk; Dose Adjustments During Pregnancy and the Postpartum Period; Maternal Adverse Reactions; Fetal/Neonatal Adverse Reactions; and Labor/Delivery.
- 8.2 Lactation—This subsection contains information about clinical considerations such as minimizing exposure and monitoring for adverse reactions. Some other areas of information that can belong in this subsection include the presence of the drug in human milk and effects of the drug on the breastfed child.
- 8.3 Females and Males of Reproductive Potential—This subsection provides information on pregnancy testing, contraception, and infertility. In information on infertility, a cross-reference to the carcinogenesis, mutagenesis, impairment of fertility subsection of the Nonclinical Toxicology section, can be made. Even when the data from the animal studies do not raise concern with respect to human fertility and/or loss effects, such information should be described in the Carcinogenesis, Mutagenesis, Impairment of Fertility subsection.

## Use of Modeling in Regulatory Submissions

**Figure 1:**
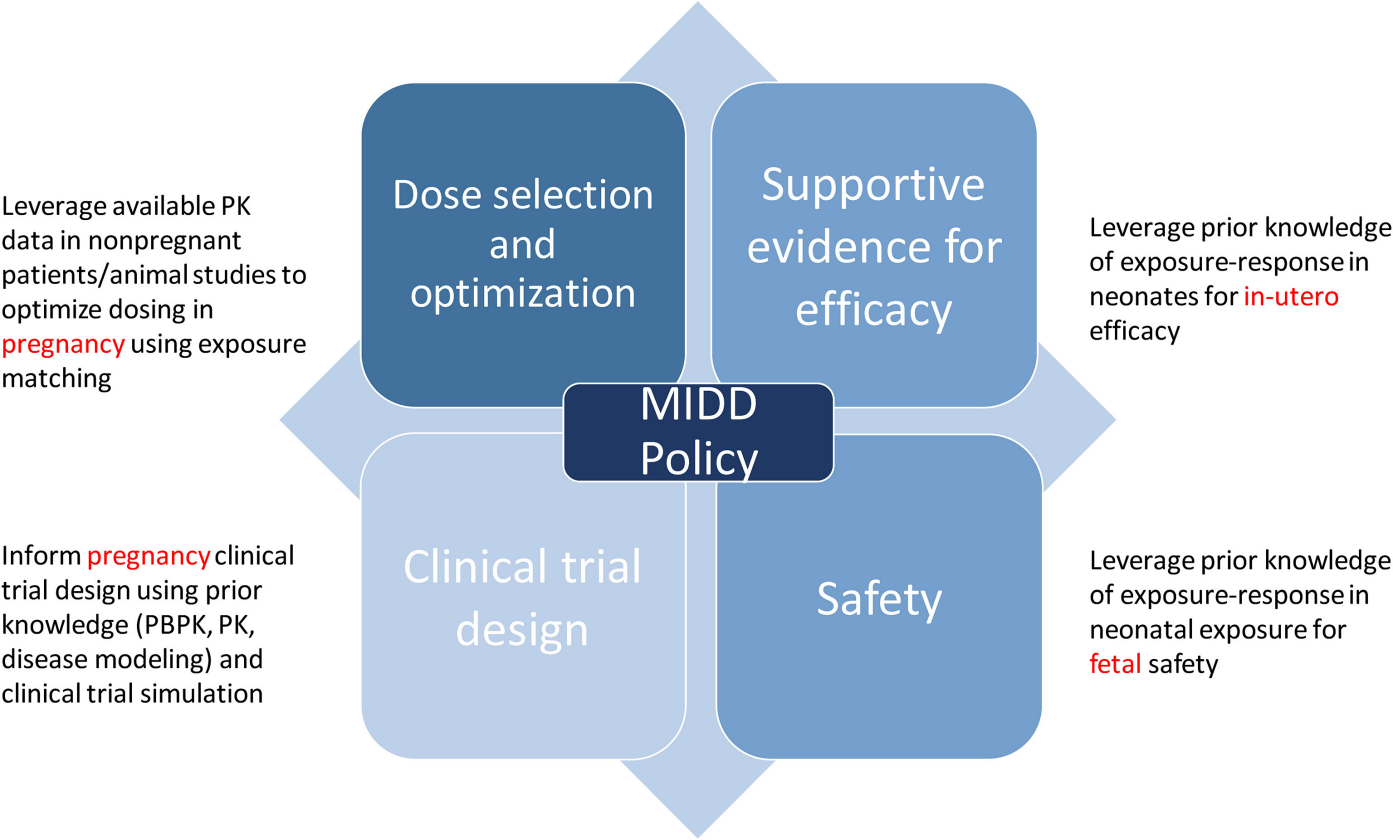
Regulatory application of model informed drug development.

The use of quantitative models that leverage our understanding of physiology, disease processes, and pharmacology are routinely applied to inform drug development. Model-informed drug development (MIDD) was formally recognized as an important application for drug development and included in the Prescription Drug User Fee Act (PDUFA) reauthorization performance goals and procedures for 2018 through 2022 (PDUFA VI) (35). The regulatory application of MIDD is broadly classified into four categories: dose optimization, supportive evidence for efficacy, clinical trial design, and safety, all of which inform policy (Figure 1) (36).

Given that there is a finite number of dosing regimens that can be formally evaluated in clinical efficacy trials, dosing regimen optimization can often be informed by modeling and simulation strategies [e.g., through nonlinear mixed effect population PK and exposure-response (ER) analyses]. MIDD is also useful in dose optimization of subgroups where therapeutic dose individualization is needed e.g., pediatrics, pregnancy, or extremes of body weight. In such conditions, model based analyses such as PBPK models and population PK models can be used to derive dosing regimens for these specific subgroups with the goal of matching the safe and effective exposure achieved in the reference patient group under the proposed dosing regimen that was studied in the efficacy and safety trials. This strategy relies on the assumption that the ER relationships for both efficacy and safety are similar between the reference group and the specific subgroups. MIDD is also useful to address complex questions regarding efficacy of drugs based on established exposure (dose)-response relationships. It allows for improving clinical trial efficiency during early phases of drug development through modeling and simualtion to determine dose selection, patient selection, trial duration and trial design. In its April 17, 2018 Federal Register Notice, FDA announced a MIDD meeting pilot program to facilitate the development and application of exposure-based, biological, and statistical models derived from preclinical and clinical data sources. The MIDD pilot program is designed to provide a process for drug developers and FDA to discuss the application of MIDD approaches, including PBPK modeling and simulation, to a specific drug development program (37). The goals of including MIDD in PDUFA VI are reducing uncertainty and attrition in drug development, providing a regulatory pathway forward for practically challenging drug development contexts, and informing appropriate use of a drug once approved.

A PBPK approach enables integration of physiologic, chemical, and drug-dependent preclinical and clinical data to model an investigational drug's ADME to generate initial PK parameters and leverage their use in subsequent simulation of untested clinical scenarios (38, 39). Currently, most applications of PBPK in regulatory decision making are limited to drug–drug interactions and initial clinical trial design. Active research is being conducted to further explore the utility of PBPK modeling in other areas to potentially expand the scope of PBPK applications (40). Pediatric PBPK models have generated attention in the last decade, because physiological parameters for model building are increasingly available and regulatory guidelines require pediatric studies during drug development.

The use of modeling and simulation to optimize design of “first-in-pediatric” PK, safety and efficacy clinical studies has increased. PBPK models have the potential to replace or inform clinical studies in children (40). Currently, the main intended application of a pediatric PBPK model is to propose an initial dosing recommendation for clinical trials at the investigational new drug (IND) application stage. PBPK/PD modeling may also provide a quantitative assessment of assumptions supporting pediatric extrapolation and pediatric trial design (41). Some researchers have suggested that for children younger than 2 years of age, the PBPK approach for predicting PK may be preferred over an allometric scaling approach in cases where ontogeny is an important determinant of drug's ADME (42, 43).

Recent studies have demonstrated the potential utility of PBPK for assessing fetal concentrations from maternal concentrations (44, 45). These PBPK assessments can be extended to assessing neonatal blood concentrations from drugs administered to mothers, which also serves as verification of the fetal model (46).

In summary, quantitative models may help provide insight on safety and efficacy to inform innovation, policy, and ultimately benefit the patient. Despite advances made in MIDD, leveraging data that are generated from all stages of drug development into appropriate modeling and simulation techniques that inform decisions remains challenging, especially in special populations. Additional discussions regarding the application of quantitative modeling approaches to drug development decisions, such as through the MIDD pilot program, may be crucial for both the sponsor(s) and regulatory review teams. As the use of MIDD by regulators and industry expands, standards and best practices must be developed to establish when and where MIDD can be applied, and what methods are appropriate in disparate settings.

## Conclusions

The need for maternal and fetal studies has now been established, and regulatory approaches are catching up quickly. Ethical considerations and FDA guidances have now established the need to include pregnant women in drug development studies when appropriate, and these studies will allow an assessment of the drug therapy in fetuses using modeling. PBPK modeling for the prediction of fetal drug concentrations is being explored in preliminary studies, and this approach is expected to mature quickly.

Science always should drive regulatory approaches. The additional needs to advance the science of fetal pharmacology are obvious and were clearly stated by Sumner Yaffe 55 years go: “Hopefully, the descriptive phase of research will be supplanted by a more sophisticated molecular approach. Only in this way will drug administration during the perinatal period truly represent optimal therapeusis instead of dogmatic posology, and contributions to a better understanding of developmental physiology be made” (1). Modeling will help fetal pharmacology to quickly move into the mainstream of drug development for the benefit of pregnant women and their fetuses.

## Author Contributions

DG, KP, VB-M, DS, and GB wrote the manuscript. All authors contributed to the article and approved the submitted version.

## Author Disclaimer

The opinions expressed in this article are those of the authors and should not be interpreted as the position of the U.S. Food and Drug Administration.

## Conflict of Interest

The authors declare that the research was conducted in the absence of any commercial or financial relationships that could be construed as a potential conflict of interest.

## Publisher's Note

All claims expressed in this article are solely those of the authors and do not necessarily represent those of their affiliated organizations, or those of the publisher, the editors and the reviewers. Any product that may be evaluated in this article, or claim that may be made by its manufacturer, is not guaranteed or endorsed by the publisher.
